# The future of robotic surgery for inflammatory bowel diseases

**DOI:** 10.1016/j.amsu.2022.104476

**Published:** 2022-08-25

**Authors:** Fahad Gul, Syed Naqash Haider Kazmi, Khawar Abbas, Sajeel Saeed, Jawad Basit

**Affiliations:** Department of Surgery, Rawalpindi Medical University, Rawalpindi, Pakistan; Department of Medicine, Rawalpindi Medical University, Rawalpindi, Pakistan

**Keywords:** Robotic surgery, Inflammatory bowel disease, Colectomy

## 1 Introduction

Robotic surgery has emerged as an effective and precise addition to minimally invasive surgical techniques which has replaced surgeons and computer-assisted mechatronic devices come in place to perform the operations. Surgery by using micro instruments, high-quality cameras, and augmented reality opens an entirely different field in terms of improving patient safety and reducing postoperative complications, and rapid return to routine life [1].

## 2 Robotic surgery and inflammatory bowel diseases

Minimally invasive techniques have been in practice for the treatment of inflammatory bowel diseases (IBD) for the last two decades due to their lower rates of wound infections, anastomosis leak, and reintervention than open surgery [2]. Robotic surgery has been the latest addition to this armor due to its lower rate of conversion to open surgery compared to laparoscopy. For example, its use for proctocolectomy and ileal-pouch anal anastomosis in ulcerative colitis patients has shown lower estimated blood loss, complications, and readmission rates [3].

In ileocolic resection for Crohn's disease, robotic surgery has shorter bowel function time, lower conversion rate to open surgery, and overall lower complication rates as compared to standard laparoscopy. A hybrid approach should be implemented when it comes to complications such as abscess, fistula, or phlegmon [4]. Due to less space, lower rectal resection which is difficult to perform by conventional surgery, and bring complications can be carried out easily by robotic surgery and this can prevent nerve damage and improves cancer outcome afterward. Robotic surgery proves its mechanical and technical viability for pelvic-related inflammatory bowel disease but there are doubts about extended colectomies and proctocolectomies which need to be addressed [5].

Robotic surgery lacks eye-hand coordination and perception by the sense of touch which can be managed by training the surgeons on three-dimensional (3D) simulation systems [1]. Although this novel surgical technique takes more operative time compared to standard laparoscopy and this problem can be improved by gaining experience and surgical expertise [6,7]. The benefits of robotic surgery outweigh the laparoscopic approach calling for further prospective studies to investigate this approach [3].

## 3 Conclusion

There is growing evidence examining the effectiveness of robotic surgery in IBD, but it will take time for surgeons to familiarize themselves with this novel technique. As advances continue to develop rapidly, it will be a great addition to the surgeon's pocket to effectively deal with IBD. For now, the decision to prioritize will ultimately dwell on patient factors, preference of the surgeon, and equipment availability.

## Ethical approval

Not applicable.

## Funding

No funding is required for the study.

## Consent

Not required.

## Registration of research studies

1. Name of the registry: Not applicable
2. Unique Identifying number or registration ID: Not applicable
3. Hyperlink to your specific registration (must be publicly accessible and will be checked): Not applicable

## Provenance and peer review

Not commissioned, externally peer reviewed.

## Ethical approval

Not applicable.

## Author contribution

Fahad Gul: Study conception, write-up, critical review and approval of the final version. Syed Naqash Haider Kazmi: Study conception, write-up, critical review. Khawar Abbas: Write-up, critical review and approval of the final version. Sajeel Saeed: Critical review and approval of the final version. Jawad Basit: Critical review and approval of the final version.

## Guarantor

Fahad Gul, Department of Surgery, Rawalpindi Medical University, Block E Satellite Town, Rawalpindi, Pakistan.

Khawar Abbas, Department of Surgery, Rawalpindi Medical University, Block E Satellite Town, Rawalpindi, Pakistan.

## Consent

Not required.

## Declaration of competing interest

All authors declared no conflict of interest.
